# Ranolazine Unveiled: Rediscovering an Old Solution in a New Light

**DOI:** 10.3390/jcm13174985

**Published:** 2024-08-23

**Authors:** Giulia Azzurra De Santis, Tommaso De Ferrari, Francesca Parisi, Marco Franzino, Agustin Ezequiel Molinero, Alessandro Di Carlo, Lorenzo Pistelli, Giampaolo Vetta, Antonio Parlavecchio, Marco Torre, Matteo Parollo, Giacomo Mansi, Pietro Paolo Tamborrino, Antonio Canu, Gino Grifoni, Luca Segreti, Andrea Di Cori, Stefano Marco Viani, Giulio Zucchelli

**Affiliations:** 1 Cardiology Unit, Department of Clinical and Experimental Medicine, University of Messina, 98122 Messina, Italy; 2 Clinical Cardiology and Heart Failure Unit, Mediterranean Institute for Transplantation and Advanced Specialized Therapies (ISMETT), 90127 Palermo, Italy; 3 S.C. Cardiologia, Ospedale Sant’Andrea, 13100 Vercelli, Italy; 4 Second Division of Cardiology, Cardio-Thoracic and Vascular Department, Pisa University Hospital, 56124 Pisa, Italy; 5 Heart Rhythm Management Centre, Postgraduate Program in Cardiac Electrophysiology and Pacing, Universitair Ziekenhuis Brussel-Vrije Universiteit Brussel, European Reference Networks Guard-Heart, 1050 Brussels, Belgium

**Keywords:** arrhythmias, ranolazine, atrial fibrillation, ventricular arrhythmias, sodium current inhibition

## Abstract

Ranolazine is an anti-anginal medication that has demonstrated antiarrhythmic properties by inhibiting both late sodium and potassium currents. Studies have shown promising results for ranolazine in treating both atrial fibrillation and ventricular arrhythmias, particularly when used in combination with other medications. This review explores ranolazine’s mechanisms of action and its potential role in cardiac arrhythmias treatment in light of previous clinical studies.

## 1. Introduction

Ranolazine is a piperazine derivative that inhibits sodium and potassium ion channel currents. The first studies on ranolazine date back to the early 1990s [1]. Even after more than thirty years, some effects of this drug are still not completely understood. Originally studied as an anti-anginal drug, ranolazine is currently approved in Europe and the United States as a second-line drug for patients with chronic coronary syndrome who show symptoms of angina refractory to other anti-anginal agents, such as beta-blockers, CCBs, and/or long-acting nitrates (COR IIa, LOE B for 2019 ESC guidelines, COR 1, LOE B for 2023 AHA/ACC guidelines) [2,3]. Early in its development, the promising antiarrhythmic potential of ranolazine became evident, revealing a broader therapeutic horizon beyond its initial anti-anginal application. Ranolazine has been investigated as an antiarrhythmic in atrial and ventricular arrhythmias, structural heart disease, heart failure, ischemic heart disease, and channelopathies. This review aims to comprehensively resemble ranolazine’s electrophysiologic effects, shedding light on its possible antiarrhythmic role in different clinical scenarios.

## 2. Side Effects

Ranolazine reaches its peak concentration within 2 to 6 h after oral intake, with metabolism predominantly hepatic via CYP3A4 [4]. Thus, attention should be paid in administering ranolazine together with CYP3A inhibitors. For the same reason, in Child–Pugh A and B patients, ranolazine plasma levels have been shown to be, respectively, 30% and 60% higher compared to patients with normal liver function; consequently, QT prolongation is enhanced in these patients. Excretion is 75% renal, which is why drug adjustments are required for eGFR below 60 mL/min, and ranolazine is contraindicated in patients with severe renal impairment (eGFR < 30 mL/min) [5].

While there are no data on ranolazine intake during pregnancy or breastfeeding, it is currently contraindicated to administer ranolazine to pediatric patients. Principal contraindications to ranolazine and the most commonly reported side effects are summarized in Table 1 [6,7,8,9]. QT prolongation is possible with ranolazine, and the careful monitoring of the QT interval is recommended for patients taking the drug. However, the effect of ranolazine on the QT interval appears to be limited, which is why it is advisable to start with a lower dose. Neurological effects and cases of myopathy have been anecdotally described but appear to be dose-dependent and remit upon discontinuation [8,9].

## 3. Ranolazine and APD

In the healthy heart, sodium channels briefly open and rapidly inactivate, producing the peak INa which dominates phase 0 of the cardiac action potential. However, the late sodium current (late INa) persists longer and contributes to the sustenance of phases 2 and 3 of the cardiac action potential. In normal ventricular myocytes, late INa constitutes only about 1% of peak INa’s amplitude [10,11]. Nonetheless, in pathological conditions, such as ischemia, myocardial infarction, heart failure, cardiac hypertrophy, and long-QT syndrome, late INa contribution to the action potential become more relevant [10,12,13,14].

In atrial cells, ranolazine partially inhibits peak INa [15,16]. This is related to a more depolarized resting membrane and to a slower phase 3 of the action potential compared to ventricular myocytes, resulting in a selective antiarrhythmic effect on atrial myocytes. As such, in the atrium, ranolazine inhibits both the early and peak sodium channel current (peak INa).

In ventricular myocytes, ranolazine primarily inhibits the late phase of the inward sodium current (late INa) as well as the rapidly activating delayed rectifier potassium current (IKr).

The inhibition of IKr in ventricular myocites explains why some QTc interval prolongation have been sometimes observed [17,18].

Thus, ranolazine’s effects on action potential duration (APD) are tissue-specific, depending on the interaction with rapid and late INa currents and IKr current. In Purkinje fibers and ventricular midmyocardial cells (M cells), in which late INa is significant, ranolazine shortens APD. Conversely, in epicardial cells, it extends APD, which leads to a decrease in the transmural dispersion of repolarization (TDR) [19].

At therapeutic levels, ranolazine prolongs APD in the atria. When late INa and APD are prolonged, as in the hypertrophic left ventricle, or in other conditions, such as heart failure and long-QT syndrome-3, ranolazine reduces APD. In atrial myocytes, ranolazine extends the effective refractory period (ERP), heightens the diastolic threshold of excitation, and lowers conduction velocity. ERP extension results from both APD prolongation and the development of post-repolarization refractoriness (PRR) [20,21,22]. These effects are attributable to the selective inhibition of peak INa by ranolazine in atrial cells. 

**Figure 1:**
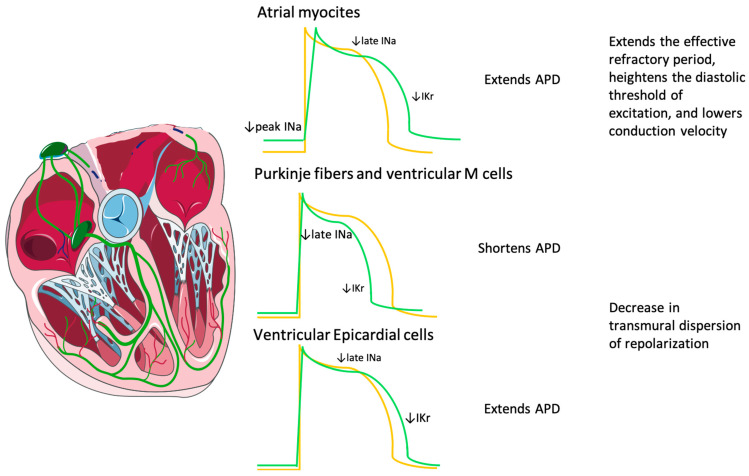
Summary of ranolazine tissue-specific effects on APD. Ranolazine’s effects on action potential are tissue-specific (green curves), depending on the interaction with rapid INa, late INa, IKr current, and their different representation on cardiac cells. APD: action potential duration; INa: inward sodium current; IKr: inward rectifier potassium current; M cells: midmyocardial cells; orange curve: standard action potential; green curve: ranolazine effect on action potential.

Figure 1 summarizes Ranolazine tissue-specific effects on APD.

## 4. Atrial Fibrillation

The electrophysiology of atrial myocytes differs significantly from ventricular myocytes and Purkinje cells, being characterized by a shorter APD, particularly in its early phases (APD50-75) [16,23].

This difference relies on specific Na^+^ channel distributions: atrial myocytes predominantly express peak Na^+^ channels over late Na^+^ channels.

Therefore, at normal therapeutic concentrations (6–10 μM), ranolazine, by blocking late INa channels, would be expected to slightly shorten the APD, potentially favoring AF rather than opposing it. However, several critical aspects should be considered.

(1) INa blockers, like ranolazine, also have a non-negligible effect on other channels, such as IKur, which are exclusively expressed in atrial cells. The blockage of Ikur current prolongs APD and atrial refractoriness.
(2) Ranolazine’s inhibitory effect on rapid Na current becomes more relevant at higher frequencies (i.e., with shorter CL). In canine-model experiments reported by Antzelevitch, ranolazine reduces sodium current Vmax by 60% with a cycle length of 300 ms. This is a common characteristic of drugs that inhibit Na channels with rapid unbinding kinetics, such as amiodarone and vernakalant.

**Figure 2:**
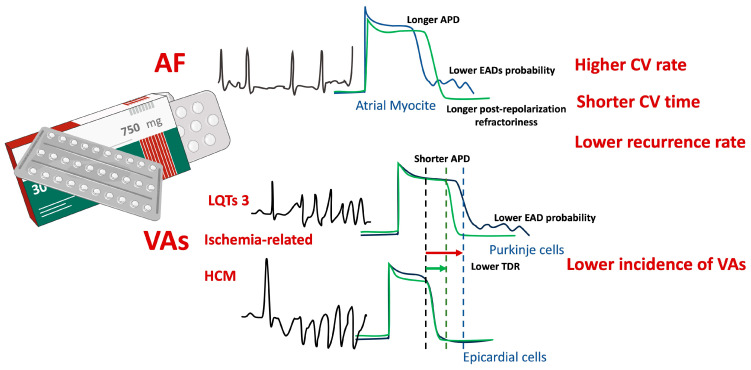
Ranolazine antiarrhythmic effect according to arrhythmic setting (atrial fibrillation and ventricular arrhythmias). AF: atrial fibrillation; APD: action potential duration; EAD: early after depolarization; CV: cardioversion; LQTs: Long QT Syndrome; VA: ventricular arrhythmia; HCM: hypertrophic cardiomyopathy; TDR: transmural dispersion of repolarization; blue curve: action potential without ranolazine; green curve: action potential under ranolazine. Red arrow: TDR without ranolazine; Green arrow: TDR with ranolazine.

Under normal heart rate conditions, ranolazine would primarily inhibit late Na current, thereby potentially preventing atrial fibrillation triggers by reducing the occurrence of atrial EADs and DADs. However, at shorter cycle lengths, its effect on late Na current becomes less significant, giving way to a rate-dependent inhibition of peak Na current. Consequently, ranolazine demonstrates an atrial-selective antiarrhythmic effect by prolonging APD through the inhibition of IKur channels and inducing a rate-related post-repolarization refractoriness via rapid peak Na channel blockade at high heart rates (Figure 2) [16,23].

Several studies have explored the potential role of ranolazine in the pharmacological cardioversion of paroxysmal AF [24,25,26]. The results across these studies are consistent, demonstrating a higher rate of sinus rhythm restoration and a shorter conversion time in patients treated with ranolazine across all ejection fraction spectra. Most of these studies compared the efficacy of adding ranolazine to amiodarone versus amiodarone alone, with reported mean times to rhythm conversion around 10–12 h in the ranolazine plus amiodarone group, significantly shorter than the conversion times reported in the literature for amiodarone alone (between 12 and 48 h) [27]. Refer to Table 2 for a detailed description of these studies.

An intriguing trial by Reiffel et al. revealed that the combination of dronedarone and ranolazine was more effective than dronedarone alone in reducing AF burden among patients with paroxysmal AF and EF > 40%. Enrolled patients were all dual-chamber pacemaker carriers, and AF burden was monitored by monthly device interrogation. To overcome the negative inotropic effect of dronedarone, reduced drug formulation was employed. A total of 131 patients were randomized to receive either placebo, dronedarone 225 mg BID only, ranolazine 750 mg BID only, or a combination of dronedarone (225 mg BID or 150 mg BID) plus ranolazine (750 mg BID). The trial concluded for the effectiveness of the combined approach in reducing AF burden vs. either placebo (*p* = 0.008), dronedarone 225 mg BID alone (*p* = 0.002), or ranolazine750 mg BID alone (*p* = 0.049) [28].

**Table 2:** Characteristics of reported studies which investigated role of ranolazine in AF. AF: atrial fibrillation; R: ranolazine; D: dronedarone; A: amiodarone; Conv.: conversion; SR: sinus rhythm; pAF: paroxysmal AF; perAF: persistent AF; NSTE-ACS: non-ST elevation-acute coronary syndrome; I.V.: intravenous; CCBs: calcium blockers; AAD: antiarrhythmic drug.

Author Year	Setting	Comparison	Other AADs	Result [*p* Value]	Side Effects
Fragakis 2012 [29]	Conv. in recent-onset AF	R (1500 mg) + A (loading dose + maintenance) vs. A alone (loading dose + maintenance)	Beta-Blocker (55%) CCBs (20%) Digoxin (14%)	Same conv. rate [0.06] Shorter time to conv. with R [<0.01]	Not reported
Koskinas 2014 [25]	Conv. in recent-onset AF	R (1500 mg) + A (loading dose + maintenance) vs. A alone (loading dose + maintenance)	Beta-Blocker (75%) CCBs (21%) Digoxin (21%)	Higher conv. rate with R [0.02]	Dizziness and nausea (2%)
Scirica 2015 [30]	AF in NSTE-ACS	I.V. R (bolus + maintenance up to 96 h) followed by R 1000 mg p.o. BID vs. Placebo	Beta-Blocker (89%)	Reduction in AF burden in the year following ACS in R group [0.01]	Not reported
De Ferrari 2015 [31]	AF recurrences after SR restoration in perAF	Placebo vs. R (375 mg) vs. R (500 mg) vs. R (750 mg)	Beta-Blocker (41%) Digoxin (13%) Previous Class I/III AAD (18%)	Same AF recurrence rate with R 500 mg and 750 mg vs. placebo [0.053]	Not reported
Reiffel 2015 [28]	pAF	Placebo vs. R (750 mg) + D placebo vs. R placebo + D (225 mg) vs. R (750 mg) + D (150 mg) vs. R (750 mg) + D (225 mg)	-	Lower AF recurrence rate with R + D 225 mg [<0.01]	Dizziness, constipation, Nausea
Tsanaxidi 2017 [25]	Conv. of recent-onset (<48 h duration) AF	R (1000 mg) + A (loading dose + maintenance) vs. A alone (loading dose + maintenance)	-	Faster SR restoration [<0.01] and higher conv rate [0.02] in R group	Not observed
Simopoulos 2018 [32]	Post-CS AF	R (750 mg p.o. in 12 h) + A (500 mg po) vs. A alone (loading dose + maintenance)	Beta-Blocker (98%) Digoxin (0.2%)	Faster SR restoration with A + R [<0.01]	Not reported

The MERLIN TIMI trial investigated the role of ranolazine in preventing AF. The study enrolled 6560 patients hospitalized with acute coronary syndrome without ST-elevation. The study conducted by Scirica et al. was a secondary analysis of the MERLIN-TIMI 36 trial, which was a large, randomized, double-blind, placebo-controlled clinical trial that assessed the efficacy of ranolazine in patients with non-ST elevation-acute coronary syndromes (NSTE-ACSs). Scirica et al. focused on analyzing the incidence of atrial fibrillation among the 6560 patients with NSTE-ACS enrolled in the MERLIN-TIMI 36 trial. The follow-up period for this study was 12 months, during which patients were continuously monitored for atrial fibrillation and other cardiovascular events. The primary endpoint of this secondary analysis was the incidence of new-onset atrial fibrillation or recurrent atrial fibrillation in patients treated with ranolazine compared to those receiving a placebo. Secondary endpoints included evaluating the impact of ranolazine on other arrhythmias and its overall safety profile in the context of atrial fibrillation management. The study found that ranolazine significantly reduced the incidence of atrial fibrillation compared to placebo. The results showed a 30% reduction in the risk of new or recurrent AF episodes in patients treated with ranolazine. Among the 3162 patients treated with ranolazine, fewer episodes of VT lasting >8 beats, and fewer episodes of supraventricular tachycardia were observed (*p* = 0.001 for both outcomes). Although not statistically significant (*p* = 0.08), a lower rate of AF was observed in the ranolazine treated arm [30].

A subsequent sub-analysis provided further insights, showing that patients in the ranolazine group had permanent AF at the time of randomization more frequently, while, when considering only patients with paroxysmal AF, AF burden was significantly lower in the ranolazine treated group (4.4% vs.16.1%, *p* = 0.015) [30].

With respect to other studies, the MERLIN TIMI trial also enrolled permanent (chronic) AF patients. Additionally, no information about other co-administered antiarrhythmic drugs (except for BBlockers) was provided.

According to studies by Antzelevitch et al., ranolazine’s ability to inhibit peak Na current appears to be diminished in the remodeled atria, raising questions about its effectiveness as an antiarrhythmic agent in cases of persistent (or chronic) AF. However, the findings of Murdock et al. regarding ranolazine’s efficacy in facilitating second electrical cardioversion (EC) in previously EC-resistant patients, especially those not receiving any class III antiarrhythmic drugs, suggest potential benefits in specific clinical scenarios, even among remodeled atria [33].

Furthermore, the synergistic effect of ranolazine when combined with class III antiarrhythmic drugs like amiodarone and dronedarone seems to be highly significant. The co-administration of amiodarone (or dronedarone) enhances the inhibition of rapid peak Na current and synergistically blocks IKur, thereby prolonging the APD. This indicates a potential maximization of ranolazine’s efficacy when used in conjunction with amiodarone or dronedarone therapy [19].

The RAFFAELLO trial tested the efficacy of ranolazine in preventing and delaying AF recurrences among patients with persistent AF who underwent sinus rhythm restoration after electric cardioversion. This study enrolled a total of 241 patients. Enrolled patients were randomized to receive either placebo or ranolazine 375 mg BID, 500 mg BID, or 750 mg BID. Most participants were already on antiarrhythmic therapy or beta-blockers at the start of the study. The trial failed in proving ranolazine effectiveness; however, the findings should be interpreted according to the above observations, which possibly explain the results. Not statistically significant differences were noted as the primary outcome of the study (time to first AF recurrence), and only a trend towards a lower rate of recurrences among the composite group of ranolazine 500 mg BID or 750 mg BID with respect to placebo emerged from the analysis. The two higher doses combined showed a relative risk of a recurrence of AF of 0.74 (95% CI 0.50–1.03, *p* = 0.053) compared with the placebo group and of 0.73 (95% CI 0.51–1.00, *p* = 0.035) compared to the 375 mg group [31].

It is worth noting that the antiarrhythmic effects of ranolazine were shown starting from concentrations slightly below 10 μM, necessitating medium to high drug doses to reach antiarrhythmic effectiveness [34]. In line with experimental models, studies reported an arrhythmic effectiveness of ranolazine when administered at doses above 1 g/die (i.e., at least 500 mg BID). Consistent with this observation, the only trend towards ranolazine effectiveness in the RAFFAELLO trial was shown when placebo was compared to the administration of medium or high drug doses [31].

Importantly, none of the above-mentioned studies reported ranolazine-related side effects, showing the extremely high tolerability and safety profile of this drug.

## 5. Ventricular Arrhythmias

The blockade of the late Na current leads to decreased Na concentrations during phases 2 and 3, which, in turn, reduces the activation of the sodium–calcium exchangers (NCXs) in the retrograde mode. This results in decreased intracellular calcium levels, shortened APD, and reduced likelihood of early afterdepolarizations (EADs) [29,32,34,35].

Notably, late Na channels are more represented in Purkinje and mid-myocardial cells compared to epicardial cells. This differential expression justifies the APD reduction in Purkinje and mid-myocardial cells following the administration of ranolazine. The selective reduction in APD determines a reduction in the transmural dispersion of repolarization (TDR) and consequently allows an antiarrhythmic effect. While the use of ranolazine in preventing EADs may seem counterintuitive in light of its inhibitory effect on the delayed rectifier potassium current IKr, studies have demonstrated its efficacy in this setting, as discussed above [19,29,31,32,34,35,36].

Antiarrhythmic effects of ranolazine in ventricular arrhythmias have been largely investigated, but robust evidence is still lacking (Table 3) [37]. The MERLIN TIMI trial stands out as the largest study to evaluate ranolazine’s efficacy in protecting against ventricular arrhythmias [30]. Ranolazine was administered intravenously and then orally. A significantly lower rate of NSVT (*p* < 0.001) and a not significant lower rate of polymorphic VT (1.2% vs. 1.4%, *p* = 0.4) were observed in patients receiving ranolazine, while no difference was found in rates of sustained VT (0.44% in both groups, *p* = 0.98). Importantly, the small number (14 patients who experienced sustained VT in each group) may have affected this result.

The RAID trial, aiming to assess the efficacy of high doses of ranolazine (1 g BID) in a high arrhythmic risk population (ICD carriers in secondary prevention or those at high risk of VA based on baseline characteristics) with ischemic or nonischemic cardiomyopathy, did not reach statistical significance for its primary endpoint (occurrence of VT or VF requiring ICD therapy or death) despite a trend favoring ranolazine (34.1% vs. 39.4% in the ranolazine and placebo groups, respectively; *p* = 0.117) [36].

A slight reduction in VT or VF recurrences requiring ICD therapy was noted among subjects treated with ranolazine. However, this study was underpowered due to high dropout rates and lower-than-expected enrolment, and ranolazine’s effects were assessed in a highly unselected sample, regardless of the underlying cardiac disease. This is particularly significant when considering a subanalysis of the RAID trial, which revealed that ranolazine significantly reduced ventricular arrhythmic events in ischemic patients but not in those with other structural heart diseases [38,39]. Also, the RYPPLE randomized cross-over trial showed a clear benefit in arrhythmic prevention among patients with coronary artery disease, although the study is based on a limited population of 105 patients and has a short follow-up period of only 30 days [40]. Factors associated with ranolazine’s effectiveness included the absence of AF and the absence of other antiarrhythmic drugs, suggesting a lower efficacy of ranolazine in more advanced arrhythmic conditions [39]. Ranolazine may exhibit greater effectiveness in preventing arrhythmias when the underlying mechanism is triggered activity or increased transmural dispersion of repolarization (TDR), while it is likely to offer limited benefits in the presence of an established arrhythmic substrate with pre-defined circuits.

A statistically significant difference was observed between arrhythmias in cardiac resynchronization therapy (CRT) carriers versus non-carriers, suggesting ranolazine’s protective effect among CRT carriers. Epicardial coronary sinus stimulation could potentially increase TDR, thereby promoting arrhythmias [39]. Ranolazine’s reduction in TDR by shortening APD in Purkinje and mid-wall cells could mitigate this effect, supporting the intriguing findings by Younis et al. [39]. However, small-sample studies have contradicted the hypothesis of a limited ranolazine efficacy in advanced disease state, demonstrating a reduction in the arrhythmic burden among patients with ischemic and nonischemic cardiomyopathy refractory to other AADs who were treated with ranolazine [41,42].

In summary, while there is inconsistency between studies regarding ranolazine’s effectiveness in the context of VA in general, from a pathophysiological point of view, it seems reasonable to consider its potential benefits in specific subsets where TDR and EADs play a central and critical role, such as in LQTS and HCM. 

A potential, though hypothetical, application of ranolazine in arrhythmic mitral valve prolapse (AMVP) is intriguing. In experimental models, stretch-induced Na channel activation can lead to calcium overload and EADs, triggering ventricular arrhythmias [43,44,45]. Ranolazine has been shown to mitigate this effect in the context of AMVP, where continuous stretches from prolapsing leaflets could promote EADs and trigger potentially fatal arrhythmias [46,47]. Given a small case series that demonstrated the potential benefit of flecainide in AMVP patients, the rationale for using ranolazine appears reasonable [46,47,48].

**Table 3:** Characteristics of reported studies which investigated role of ranolazine in ischemic-related VA. VT: ventricular tachycardia; R: ranolazine; A: amiodarone; NSTE-ACS: non-ST elevation-acute coronary syndrome; IV: intravenous; BID: bis in die; AAD: antiarrhythmic drug; ICD: implantable cardioverter-defibrillator; GI: gastrointestinal; VA: ventricular arrhythmia; CAD: coronary artery disease; CRT: cardiac resynchronization therapy; AF: atrial fibrillation.

Author	Setting	Comparison (Numerosity)	Other AADs	Ranolazine Dose	Result [*p* Value]	Side Effects
Scirica 2007 [49]	VT in NSTE- ACS	R (3162) vs. Placebo (3189)	Beta-Blocker (89%)	I.V. R (bolus + manteinence) and subsequent po BID administration	Lower incidence of VT in R group	Not reported
Bunch 2011 [41]	AADs refractory VT with recurrent ICD shocks	(12)	A (92%) Lidocaine/Mexiletine (50%) Sotalolo (8%)	500 mg daily	Reduction in both VT burden ICD shocks	GI problems (17%) Severe hypoglycemia (8%)
Pelliccia 2015 [40]	Palpitations in stable CAD	R (53) vs. Placebo (52)	Beta-Blocker (100%)	750 mg BID	R more effective in reducing both VA and symptoms in R group	Not observed
Curnis 2017 [42]	ICD carriers with increased VA burden/ at least one appropriate ICD intervention in the previous 6 months	(17)	Beta-Blocker (94%) A (71%)	500 mg BID (dose reduction to 350 mg BID allowed)	Reduction in both VA/ICD interventions [0.045] and symptoms [<0.01]	Not observed
Zareba 2018 [38]	High-risk ICD carriers	R (510) vs. Placebo (502)	Beta-Blocker (93%) Digitalis (17%) A (10%) Other antiarrhythmic (17%)	500 mg BID (7 days) followed by uptritation to 1000 mg BID	R more effective in reducing recurrent VT/VF requiring ICD therapy [0.03]	Dizziness (8%) Nausea (6%) Costipation (4%) vomiting (2%) headaches, and fatigue (2%) Indigestion, cough, chest pain, shortness of breath, and dry mouth (1%)
Younis 2022 [39]	High-risk ICD carriers	R (510) vs. Placebo (502)	Beta-Blocker (93%) Digitalis (17%) Amiodarone (10%) Other antiarrhythmic (17%)	500 mg BID (7 days) followed by uptritation to 1000 mg BID	Ranolazine more effective in reducing VA burden, especially in CRT-treated patients, in patients without AF and AADs naïve [<0.01]	Not observed

### 5.1. LQTS

While only preclinical studies explored the potential use of ranolazine in Long QT Syndrome type 2, more evidence exists regarding its application in Long QT Syndrome type 3 (LQTS3) [50]. LQTS3 is characterized by a gain-of-function mutation in the SCN5A gene, leading to a threefold increase in late Na current, which significantly prolongs the APD and promotes EADs. Two notable small-scale studies by Chorin and Moss evaluated ranolazine’s ability to inhibit the late sodium current and its utility in LQTS3 (Table 4) [50,51].

The study by Moss et al. demonstrated a concentration-dependent shortening of the QTc interval (by approximately 26 ± 3 ms, *p* < 0.0001) and improvement in left ventricular relaxation following ranolazine infusion (approximately 585 mg over 8 h) [52]. Chorin et al.’s research corroborated the concentration-dependent capability of ranolazine to block SCN5A. However, it is important to note that while ranolazine showed a comparable efficacy to flecainide and mexiletine, it was found to be of poor efficacy during significant bradycardia (heart rate below 40 bpm) [51].

A metanalysis concluded for ranolazine effectiveness in shortening QTc interval in LQT3 patients at the cost of fewer side effects [51]. However, data remain exceedingly limited, and further studies are needed to validate the potential application of ranolazine in this setting.

**Table 4:** Characteristics of reported studies which investigated role of ranolazine in LQT3 and in HCM. AAD: antiarrhythmic drug; ACS: acute coronary syndrome; BID: bis in die; CAD: coronary artery disease; CCS: Canadian Collage of Cardiology; GI: gastrointestinal; HCM: hypertrophic cardiomyopathy; ICD: implantable cardioverter-defibrillator; IV: intravenous; LQTS: Long QT Syndrome, QTc: corrected QT; VA: ventricular arrhythmia; VT: ventricular tachycardia; PVC: premature ventricular complex.

Author	Setting	N°	Dose Ranolazine	Result [*p* Value]	Side Effects
Moss 2008 [52]	LQTS3 (ΔKPQ mutation)	5	IV R (bolus + maintenance)	QTc interval reduction and improvement in diastolic relaxation [<0.01]	Not reported
Chorin 2016 [51]	LQTS3	8	500 mg BID (up-titration to 1000 mg BID after 3 days)	QTc interval reduction [0.01]	Constipation (13%)
Olivotto 2018 [53]	Symptomatic non-obstructive HCM	40 (R) vs. 40 (Placebo)	500 mg BID (up-tritation up to 1000 mg BID)	Reduction in PVCs/24 h burden [0.04]	Not reported
Argirò 2023 [54]	HCM	119	375 mg BID (up-titration to 500 mg BID)	Reduction in CCS class [symptomatic relief in 73%]	GI problems, dizziness, and headache (2%) Augmented transaminase and skin rash (1%)

### 5.2. HCM

It is widely recognized that enhanced late sodium current (INaL) plays a pivotal role in the electrophysiological abnormalities of hypertrophic cardiomyopathy (HCM). Specifically, in human HCM cardiomyocytes, increased INaL activity prolongs action potentials and triggers early and delayed afterdepolarizations, which are established markers of electrical instability. Furthermore, elevated INaL results in calcium ion overload during diastole, impairing myocyte relaxation and perfusion [55]. In vitro studies with ranolazine have shown an ability to reduce diastolic disfunction and the arrhythmic propensity of HCM cardiomyocytes, suggesting potential clinical benefit [56].

On this basis, RESTYLE HCM, a double-blind trial, was designed to evaluate the impact of ranolazine on the functional capacity and diastolic function in non-obstructive HCM patients. However, the primary endpoint did not meet statistical significance, probably because of the variable expression of late sodium channels current in HCM cardiomyocytes [53]. Nevertheless, ranolazine treatment was associated with a significant reduction in the 24 h ventricular arrhythmic burden (>50%) compared to the placebo.

Real-world experience by Olivotto and Al showed ranolazine’s effectiveness as a second-line therapy in both reducing angina symptoms, which are related to wall stress and microvascular dysfunction, and ventricular arrhythmic burden (Table 4) [53].

## 6. Conclusions

While evidence supporting ranolazine effectiveness in VAs is still lacking, its use in cases of paroxysmal AF, especially when combined with a class III antiarrhythmic drug, appears to be highly effective in reducing time to cardioversion, increasing cardioversion success rate, and reducing AF recurrence. It is worth noting that while the antiarrhythmic effects of ranolazine manifest at high doses, often exceeding the maximum recommended dose of 1.5 g/day, the side effects associated with its use are substantially negligible, delineating a highly safe profile.

## Figures and Tables

**Table 1:** Ranolazine’s side effects and contraindications.

Side Effects [9]	Frequency
Dizziness	5–7%
Constipation	4–8%
Headache	6%
Nausea	2–4%
Abdominal pain	1%
Dry mouth	1%
Peripheral edema	1%
Pancytopenia	Extremely rare (<0.1%)
Neurological symptoms (including myopathy)	Anectodical
Side effects are observed more frequently in patients with eGFR between 80 and 30 mL/min/1.73 m^2^ and when a higher dosage of 1000–1500 mg twice daily is administered [5,7]	
Principal Contraindications	
Pre-existing QT prolongation [5]	
Hepatic impairment [5]	Child–Pugh C
Renal Failure [5]	eGFR < 30 mL/min/1.73 m^2^
Pediatrics	
Strong CYP3A inhibitors [4]	Ketoconazole, itraconazole, macrolides, clarithromycin, erythromycin, non-dihydropyridine calcium blockers, and ritonavir
